# A Langendorff-like system to quantify cardiac pump function in adult zebrafish

**DOI:** 10.1242/dmm.034819

**Published:** 2018-09-10

**Authors:** Hong Zhang, Alexey V. Dvornikov, Inken G. Huttner, Xiao Ma, Celine F. Santiago, Diane Fatkin, Xiaolei Xu

**Affiliations:** 1 Department of Biochemistry and Molecular Biology, Mayo Clinic, Rochester, MN 55902, USA; 2 Cardiovascular Surgery Department, the Second Xiangya Hospital of Central South University, Changsha 410011, China; 3 Molecular Cardiology Division, Victor Chang Cardiac Research Institute, Sydney, NSW 2010, Australia; 4 St. Vincent's Clinical School, Faculty of Medicine, University of New South Wales, Sydney, NSW 2052, Australia; 5 Clinical and Translational Sciences Track, Mayo Clinic Graduate School of Biomedical Sciences, Mayo Clinic College of Medicine and Science, Rochester, MN 55092, USA; 6 Cardiology Department, St. Vincent's Hospital, Sydney, NSW 2010, Australia

**Keywords:** Cardiac contractility, Cardiac pump function, Langendorff, Zebrafish

## Abstract

Zebrafish are increasingly used as a vertebrate model to study human cardiovascular disorders. Although heart structure and function are readily visualized in zebrafish embryos because of their optical transparency, the lack of effective tools for evaluating the hearts of older, nontransparent fish has been a major limiting factor. The recent development of high-frequency echocardiography has been an important advance for *in vivo* cardiac assessment, but it necessitates anesthesia and has limited ability to study acute interventions. We report the development of an alternative experimental *ex vivo* technique for quantifying heart size and function that resembles the Langendorff heart preparations that have been widely used in mammalian models. Dissected adult zebrafish hearts were perfused with a calcium-containing buffer, and a beat frequency was maintained with electrical stimulation. The impact of pacing frequency, flow rate and perfusate calcium concentration on ventricular performance (including end-diastolic and end-systolic volumes, ejection fraction, radial strain, and maximal velocities of shortening and relaxation) were evaluated and optimal conditions defined. We determined the effects of age on heart function in wild-type male and female zebrafish, and successfully detected hypercontractile and hypocontractile responses after adrenergic stimulation or doxorubicin treatment, respectively. Good correlations were found between indices of cardiac contractility obtained with high-frequency echocardiography and with the *ex vivo* technique in a subset of fish studied with both methods. The *ex vivo* beating heart preparation is a valuable addition to the cardiac function tool kit that will expand the use of adult zebrafish for cardiovascular research.

## Abstract (precis)

**Summary:** Here, we describe a new experimental tool to assess contractility of the isolated zebrafish heart perfused in a Langendorff-like system, which will facilitate using zebrafish as a model for human cardiomyopathies.

## INTRODUCTION

The zebrafish (*Danio rerio*) has emerged as a popular vertebrate model for cardiovascular research (Gut et al., 2017; Stoyek et al., 2016). Despite having only 2 chambers, zebrafish hearts have striking physiological similarities to human hearts (Becker et al., 2011; Werdich et al., 2012) and extensive genetic conservation (Bakkers, 2011; Howe et al., 2013). Historically, optically transparent zebrafish embryos have been mainly used to study heart development and congenital heart disease. The potential utility of zebrafish for study of adult-onset heart diseases is relatively underexplored. Several acquired cardiomyopathy models have been generated, including anemia-induced cardiomyopathy (Hoage et al., 2011; Sun et al., 2009), doxorubicin (DOX)-induced cardiomyopathy (DIC) (Ding et al., 2011; Packard et al., 2017) and hyperglycemia-induced cardiomyopathy (Sun et al., 2017). Recently, several studies provided proof-of-principle evidence for the value of zebrafish models for assessing the functional effects of human genetic variants (Asimaki et al., 2014; Huttner et al., 2013; Reischauer et al., 2014; Yang et al., 2016). Of 51 genes known to cause dilated cardiomyopathy, 49 (96%) have been shown to have corresponding zebrafish homologs (Shih et al., 2015), and morpholino-mediated knockdown of many of those genes causes dilated cardiomyopathy (Vogel et al., 2009). In addition to studies using embryonic zebrafish models (Bendig et al., 2006; Hassel et al., 2009; Knoll et al., 2007), studies have used adult zebrafish to find new cardiomyopathy genes through mutagenesis screening (Ding et al., 2013, 2016) and for evaluation of therapeutic strategies such as mammalian target of rapamycin inhibition (Ding et al., 2011, 2012).

The transparency of embryonic zebrafish is lost with age, and a major factor that has limited adult zebrafish studies has been the lack of effective cardiac phenotyping tools (Yang et al., 2016). In mammalian animal models, cardiac function is frequently quantified *in vivo* using ultrasound-based echocardiography. Low-frequency ultrasound transducers have not been able to adequately visualize adult zebrafish hearts because of their small size (∼1 mm in diameter). The recent development of high-frequency echocardiography (HFE) is an important technical advancement that has effectively addressed this need. With a substantially improved spatial resolution (∼30 µm axial resolution), quantification of systolic and diastolic function in adult fish is now possible (González-Rosa et al., 2014; Hein et al., 2015; Ho et al., 2002; Lee et al., 2016; Liu et al., 2013; Wang et al., 2017).

The mammalian Langendorff perfused heart is a century-old technique that allows acute pharmacologic manipulation through injection of drugs into the coronary system and direct monitoring of functional effects using a suite of parameters, including ventricular pressure and the velocity of ventricular contraction and relaxation (Langendorff, 1898; Liao et al., 2012). In echocardiography, cardiac contractile function is conventionally assessed by determining ejection fraction (EF) or fractional shortening (FS). EF is based on differences between end-diastolic and end-systolic volumes (EDV and ESV, respectively) during each cardiac cycle, and FS is derived from the single-plane end-diastolic and end-systolic linear dimensions. These indices can also be quantified in isolated heart systems without the influence of venous return, vascular tone and neurohumoral factors. This alternative cardiac phenotyping method has not previously been possible in zebrafish.

The aim of this study was to establish a Langendorff-like *ex vivo* technique for assessing cardiac pump function in adult zebrafish, building on previous reports of isolated beating zebrafish heart preparations (Hecker et al., 2008) and techniques to quantify contractility at the single-cell level (Bovo et al., 2013; Dvornikov et al., 2014). We optimized experimental conditions, adjusting parameters such as temperature, pH, beating frequency, extracellular calcium {[Ca^2+^]_o_} and mechanical load, to ensure consistent quantification of cardiac function in explanted hearts. On the basis of biplane optical imaging of beating hearts, we derived a set of cardiac functional indices, determined effects of age and sex on these indices, compared results obtained *ex vivo* with those obtained *in vivo* using HFE in the same fish, and used our new methods to define cardiac dysfunction in the DIC model (Ding et al., 2011).

## RESULTS

### Establishment of an *ex vivo* perfusion system

**Fig. 1.**
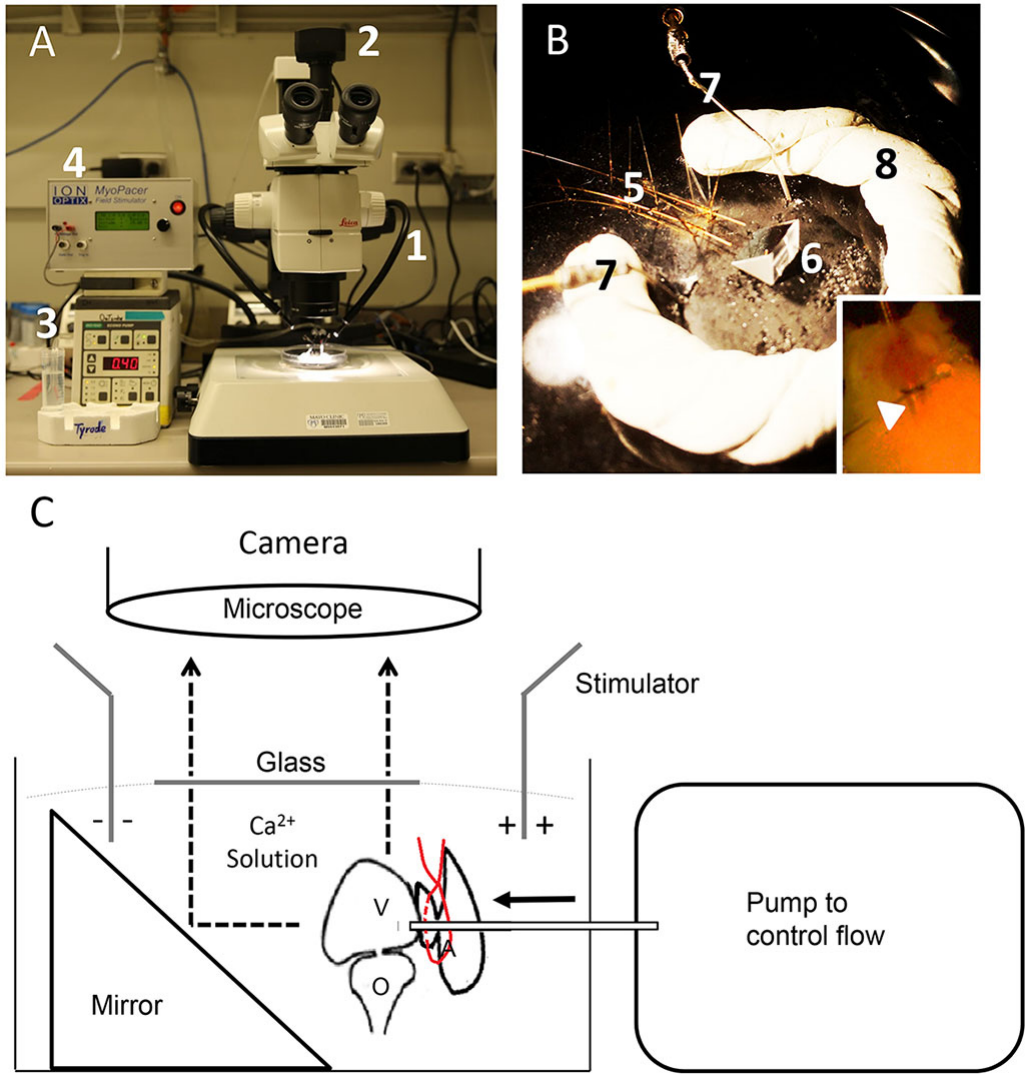
**Setup of an *ex vivo* system for an isolated zebrafish heart.** (A) Equipment needed, including a dissecting microscope (1), with a camera (2), pump (3) and stimulator (4). (B) Two-line 34G catheters (5) are placed on a Sylgard-coated Petri dish with the use of insect pins; 2 hearts are tied on the ends of the catheters (inset depicts the position of a cannula in the heart; arrowhead points to the place of the tie by the atrioventricular junction), which are put in front of a mirror (6); platinum electrodes (7) are used for electrical field stimulation. A pool (8) was made to control the solution level. (C) Diagram of the perfusion system.

To enable quantification of cardiac pump function in beating explanted adult zebrafish hearts, we developed an *ex vivo* perfusion system adapted from our previously established method of isolating membrane-intact zebrafish cardiomyocytes (Dvornikov et al., 2014), which was based on recommendations from the study by Zhang et al. (2011). Hearts were quickly isolated from anesthetized adult zebrafish, most of the atrium was excised, the atrioventricular canal was exposed, and the ventricle was then cannulated with a 34G catheter that was microsurgically sutured to the atrium close to the atrioventricular canal. The catheter tip was opened to the ventricular lumen and positioned to avoid touching the ventricular wall. There was no apparent cardiac tissue damage in the vicinity of the atrioventricular canal or evidence that the catheter was physically impeding adequate cardiac output (see Materials and Methods and Fig. 1B, inset). A peristaltic pump was used to provide constant anterograde flow of a modified Tyrode solution from the atrioventricular junction through the ventricle and to the outflow tract (OFT, bulbus arteriosus; Fig. 1). Ventricular inflation was maintained by this constant perfusion. We found that it was crucial to keep the OFT intact for maintenance of ventricular pressure and that its damage resulted in ventricular collapse (data not shown). Caution was also required to avoid touching heart tissue with surgical instruments, because even small invisible ruptures might affect ventricular contractility. A low perfusion rate of ∼0.05 ml min^–1^ was maintained initially to allow bubbles and blood clots to be washed out of the ventricle.

Cannulation of adult zebrafish hearts to attach the atrioventricular canal onto a 34G catheter using a 10-0 Prolene suture involved microsurgery, and this procedure was technically challenging. Most of the data reported were obtained from adult fish older than 3 months with a body weight (BW) more than 200 mg. However, we were able to successfully conduct the microsurgical procedures in fish as young as 1 month old with a BW of ∼70 mg.

**Fig. 2.**
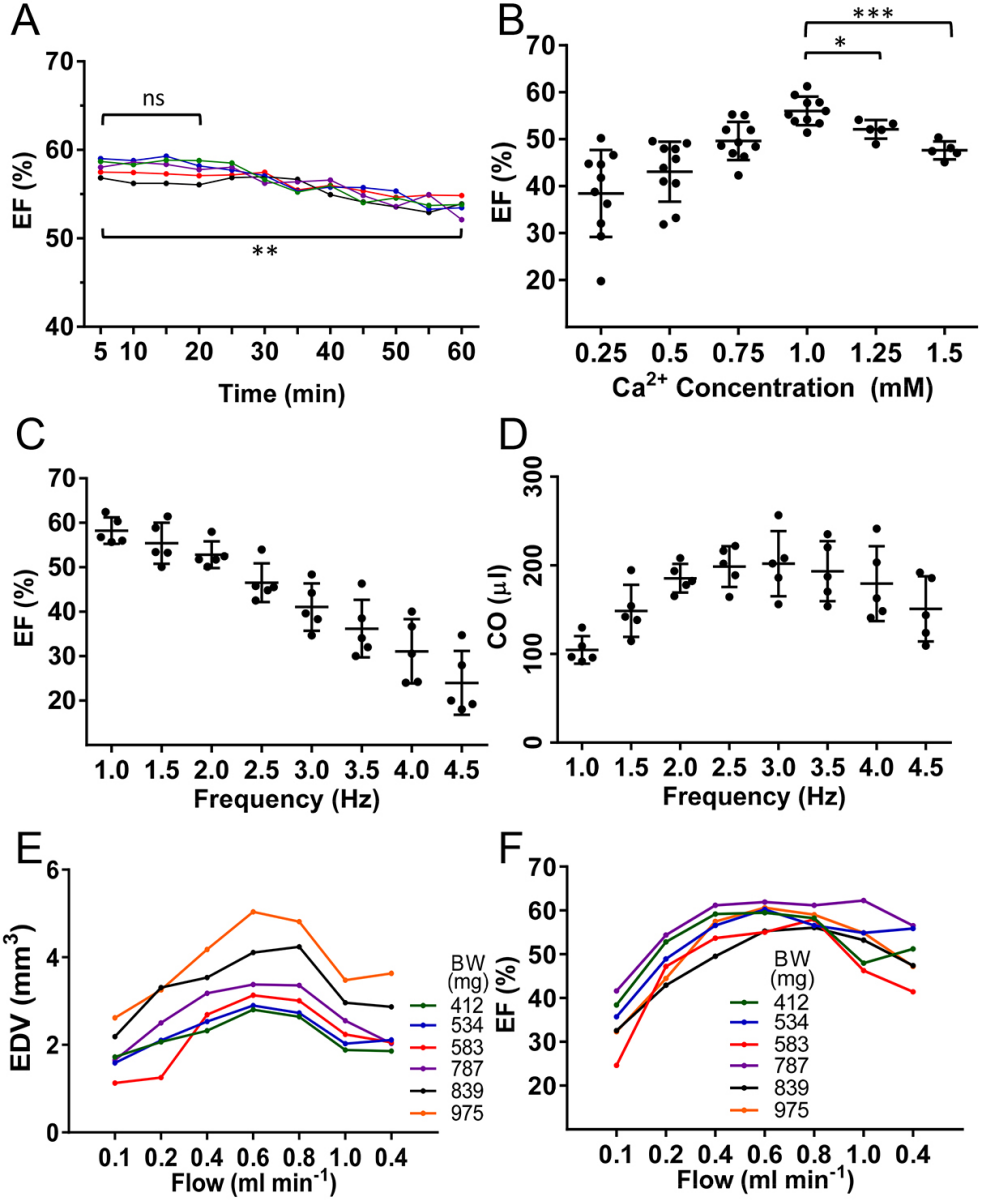
**Impact of experimental variables on cardiac pump function.** (A) Ventricular EF of adult zebrafish heart during the time window of the *ex vivo* procedure (*n*=5). (B) EF as a function of [Ca^2+^]_o_ in physiological solution (*n*=10). (C) EF as a function of pacing frequency (*n*=5). (D) Cardiac output (CO) as a function of pacing frequency (*n*=5). (E) Individual data on EDV as a function of flow rate (*n*=6). Key shows the BW (mg) of each fish. (F) Individual data on EF as a function of flow rate (*n*=6). Data are mean±s.d. Paired Student's *t*-test was used for statistical analysis. ns, nonsignificant; *P≤0.05; **P≤0.005; ***P≤0.0001.

When driven by electrical field stimulation, isolated zebrafish hearts were able to beat *ex vivo* for several hours, and pump function deteriorated only minimally in the first 30 min (Fig. 2A). A mirrored perfusion chamber was used to acquire high-speed videos of beating hearts in both transverse and longitudinal planes simultaneously (Fig. 1A,C), and this facilitated evaluation of irregularly shaped ventricles. Our dual-catheter design also enabled 2 hearts to be recorded at the same time, allowing simultaneous comparison of control and mutant hearts (Fig. 1B; Movie 1).

### Identification of factors that affect cardiac pump function *ex vivo*

In Langendorff preparations, the pump function of an isolated heart can be dramatically affected by several parameters, including time on the rig, [Ca^2+^]_o_, flow rate of the perfusing buffer and pacing frequency (Liao et al., 2012). Because factors such as the supply of energy substrates and oxygen in *ex vivo* perfused and paced hearts can affect contractile function over time, we determined temporal changes in EF in a set of hearts without any interventions and under the following conditions: 1 mM Ca^2+^, 0.4 ml min^–1^ flow rate, 2 Hz frequency (Fig. 2A). Importantly, cardiac contractility was stable during the first 20 min (*P*=0.06), when most of our experiments are completed, and EF was reduced by only 7.6% (absolute EF percentage points) after 60 min of perfusion and pacing (*P*=0.0027).

Then, to determine the impact of Ca^2+^ concentration in the Tyrode perfusion buffer, we recorded contractile function under gradually increasing concentrations of Ca^2+^ ranging from 0.25 to 1.5 mM. Surprisingly, we found that EF initially increased, peaked at 1.0 mM, and then decreased at Ca^2+^ concentrations of 1.25 mM or higher (Fig. 2B). Previously, using zebrafish ventricular cardiomyocytes, we found that increasing [Ca^2+^]_o_ concentration from 0.5 to 4 mM resulted in increased twitch forces (Dvornikov et al., 2014), but cell survival was also reduced at the higher concentrations (A.V.D., unpublished). Mammalian Tyrode buffer typically contains Ca^2+^ concentrations in a range from 1.3 to 1.8 mM. However, depending on the model system used, lower concentrations might be preferable (Dvornikov et al., 2012; Glukhov et al., 2013). In our *ex vivo* model, we found that cardiac contractile performance was best with a Ca^2+^ concentration of 1.0 mM, and this was selected as the standard Ca^2+^ concentration for all subsequent studies.

Next, we analyzed the impact of the pacing frequency on isolated heart contractility. The contractile force developed by the heart increases or decreases with the frequency of stimulation, an intrinsic property of cardiac muscle termed the force-frequency relationship. Mechanistically, the force-frequency relationship is mediated through accelerated Ca^2+^ cycling via the sarcoplasmic reticulum and myofilament sensitization to activator Ca^2+^ (Schouten and ter Keurs, 1986; Dvornikov et al., 2012). In *ex vivo* perfused and paced zebrafish hearts, we found that EF decreased with increased pacing frequency (Fig. 2C). In contrast, cardiac output, the product of stroke volume and heart rate, initially increased with pacing frequencies ranging from 1.0 to 2.0 Hz, plateaued at 2.0 to 3.5 Hz, and then decreased at frequencies from 3.5 to 4.5 Hz (Fig. 2D). Pacing frequencies more than 2 Hz result in increased diastolic Ca^2+^ levels in fish (Llach et al., 2011; Zhang et al., 2011). We decided to use 2 Hz for future standard experimentation because this frequency is still on the ascending limb of the frequency-cardiac output relationship and approximates heart rates in zebrafish at 28°C (∼120 beats min^–1^, according to our unpublished electrocardiographic data and HFE data from other groups) (Sun et al., 2017; Wang et al., 2017).

Lastly, we analyzed the impact of different mechanical loads on cardiac contractility by changing the perfusion flow rate. We found that EDV increased with flow rates from 0.1 to 0.6 ml min^–1^, and then decreased with flow rates from 0.8 to 1.0 ml min^–1^. Similarly, EF increased with flow rates between 0.1 and 0.4 ml min^–1^, plateaued with flow rates between 0.4 and 0.8 ml min^–1^, and then decreased with flow rates more than 1.0 ml min^–1^ (Fig. 2E). We considered the initial heart enlargement an indicator of the quality of heart preparation without undesired leakage or rupture. Because contractility and EDV seemed to stabilize for flow rates between 0.4 and 0.8 ml min^–1^, depending on fish body size (and heart size), we used this maximum contractility as the benchmark for cardiac function comparisons (Fig. 2F). Although these conditions represent a relatively hypercontractile state, we reasoned that this would be useful for determining maximal cardiac performance and for unmasking latent defects in heart function.

In summary, we determined the effects of time, [Ca^2+^]_o_ levels, pacing frequency and mechanical load on cardiac performance in explanted wild-type zebrafish hearts, All subsequent functional studies were conducted under the standard conditions of 1 mM [Ca^2+^]_o_, 2-Hz pacing rate and a set of increasing flow rates until reaching the maximal contractility.

### Effects of age, sex and background strain on cardiac function

**Fig. 3.**
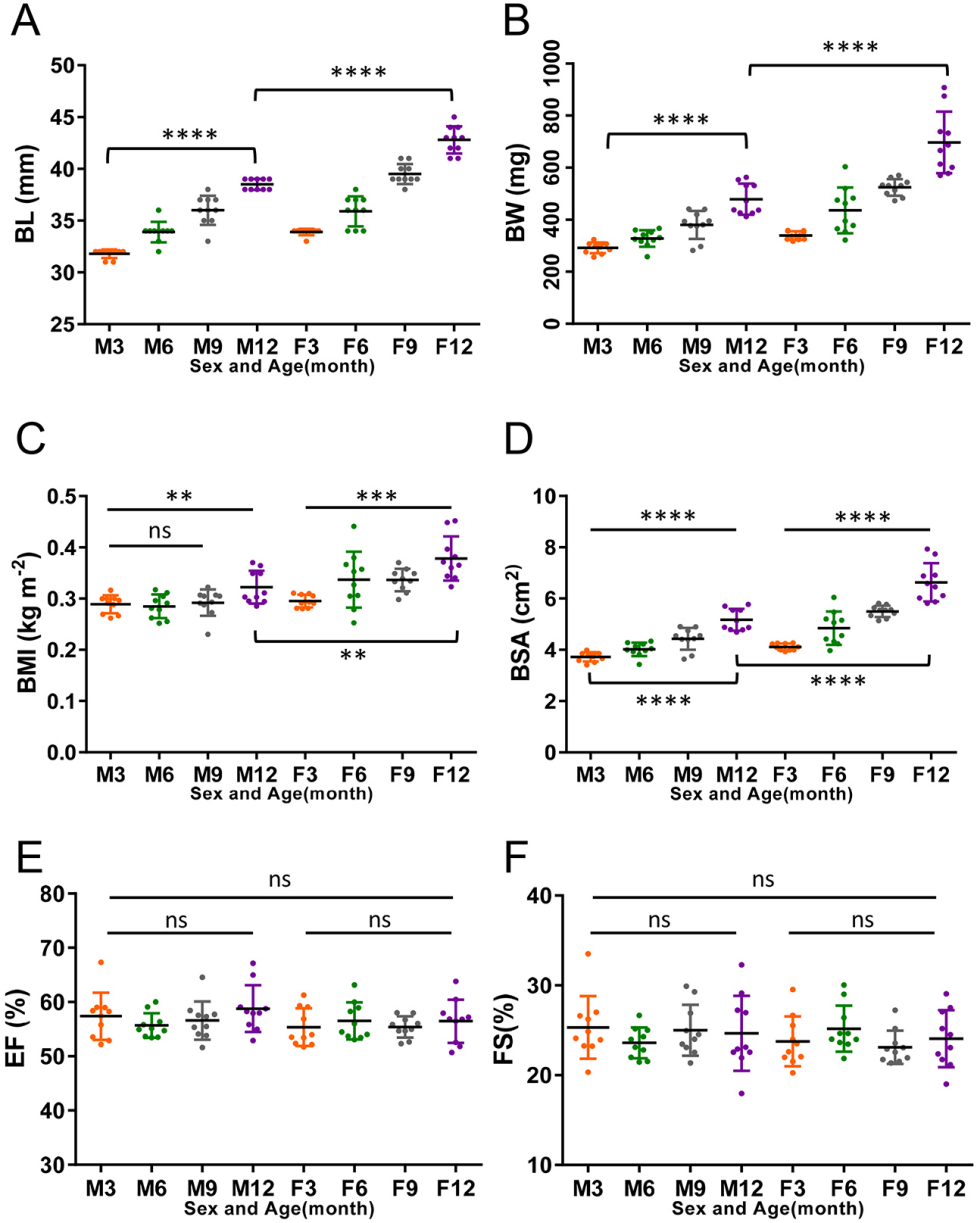
**Baseline fish exterior parameters and** **heart function in wild-type fish.** (A) Quantification of BL in male or female wild-type fish (*n*=10 for each group) at 3, 6, 9 and 12 months. (B-D) BW (B), BMI (C) and BSA (D) in the same groups of fish. (E) Quantification of EF. (F) Quantification of FS. Data are mean±s.d. For statistical analysis, unpaired Student's *t*-test was conducted for 2 groups that are connected by brackets, and one-way analysis of variance was conducted for 3 or more groups that are connected by lines. ns, nonsignificant; **P≤0.005; ***P≤0.0005; ****P≤0.0001.

Next, we applied the optimized *ex vivo* protocol to determine the baseline cardiac function parameters in wild-type fish of the WIK background strain at 3, 6, 9 and 12 months of age. In our fish facility under standard housing conditions, fish grew significantly bigger between 3 and 12 months, as measured by body length (BL), body weight (BW), body mass index (BMI) and body surface area (BSA) (Fig. 3A-D). By 12 months, female fish were significantly larger than male fish in all morphometric parameters (Fig. 3A-D). In contrast, both EF and FS were unaltered by age or sex (Fig. 3E,F, Table 1).

**Table 1.**
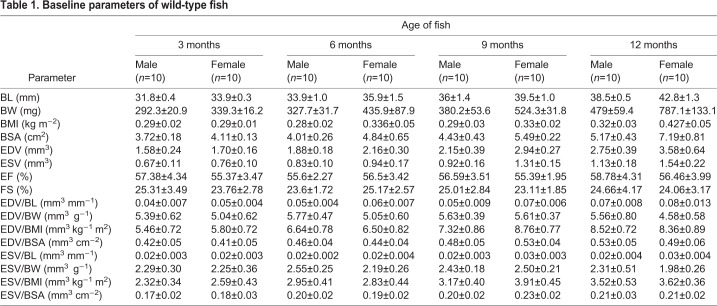
**Baseline parameters of wild-type fish**

**Fig. 4.**
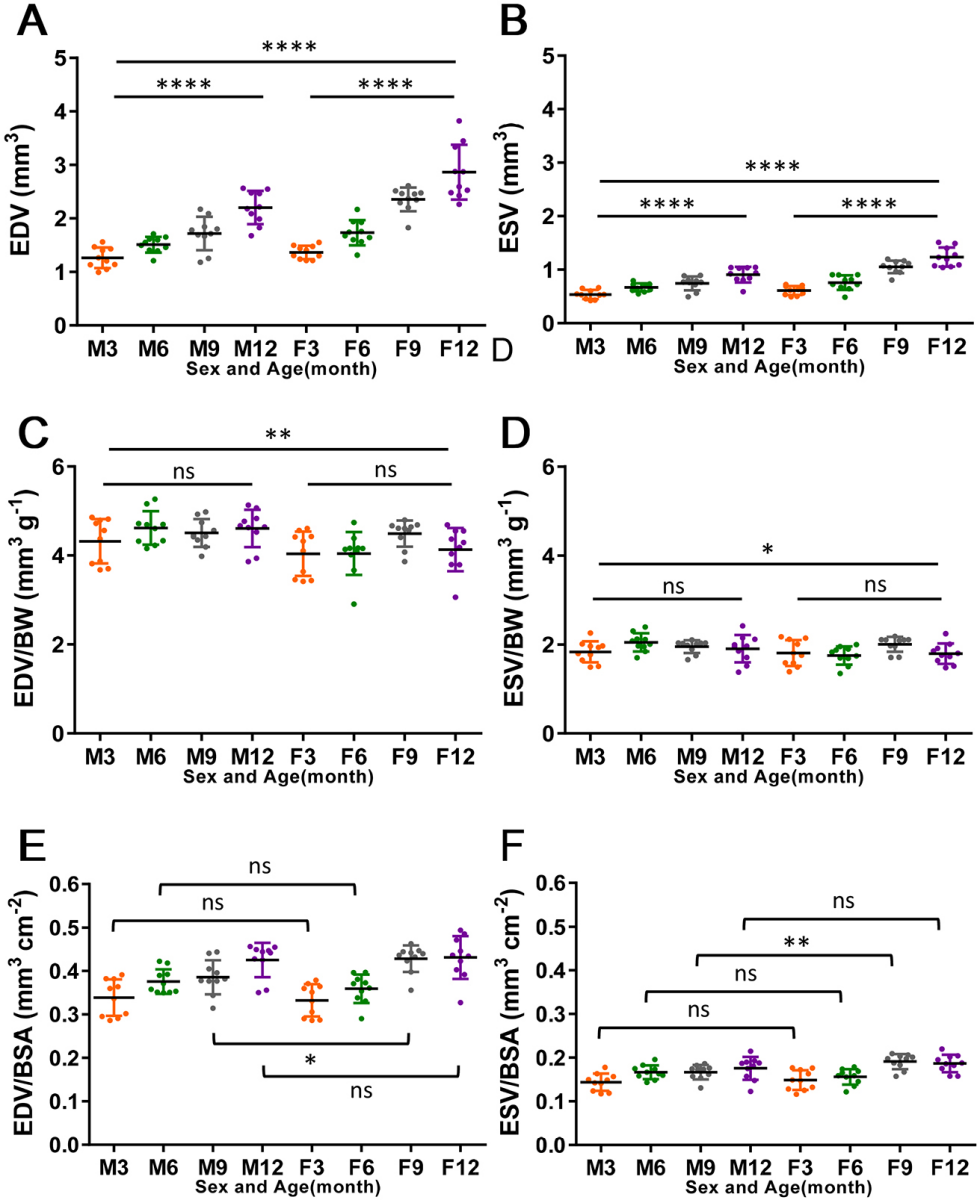
**Normalization of ventricular volumes.** (A,B) EDV (A) and ESV (B) both increase with age (3, 6, 9 and 12 months wild-type fish; *n*=10 for each group). (C,D) EDV and ESV normalized to BW: EDV/BW (C) and ESV/BW (D); neither had any dependency on age. (E,F) EDV and ESV normalized to BSA: EDV/BSA (E) and ESV/BSA (F). Data are mean±s.d. For statistical analysis, unpaired Student's *t*-test was conducted for 2 groups that are connected by brackets, and one-way analysis of variance was conducted for 3 or more groups that are connected by lines. ns, nonsignificant; *P≤0.05; **P≤0.005; ****P≤0.0001.

To calculate ventricular volumes from two perpendicular projections of a heart, we assumed a half-ellipsoid shape and used the area-length formula (Lang et al., 2005; Tortoledo et al., 1983; Wahr et al., 1983). In keeping with age- and sex-related size differences, EDVs and ESVs were significantly different between male and female fish at different time points (Fig. 4A,B). Thus, normalization of chamber size to body size was required to allow more accurate comparisons between fish. Similar to findings for HFE-determined ventricular volume measurements (Wang et al., 2017), we found that both BW and BSA were effective in mitigating body size-dependent chamber volume differences (Fig. 4C-F), but BL and BMI were less effective. Because BSA in fish is derived from BW, we chose to use BW as the normalization parameter. Given that BW in a female fish can be affected by whether eggs in her body have been laid, we recommend conducting experiments in male fish to eliminate this contributor of variation.

To investigate possible effects of genetic background on heart size and contractility, we compared 2 wild-type strains, WIK and NHGRI-1 (LaFave et al., 2014), at 6 months of age. We found no significant differences in EF, FS, EDV/BW or ESV/BW between 2 groups of the same sex (Fig. S1). To further validate volume and contractility measurements, we tested interobserver variability by asking 3 different investigators to measure EDV and ESV using the same video files. We found no significant differences between those 3 measurements or the derived EF and FS (Fig. S2).

### Comparison of cardiac parameters obtained by the *ex vivo* method and HFE

**Fig. 5.**
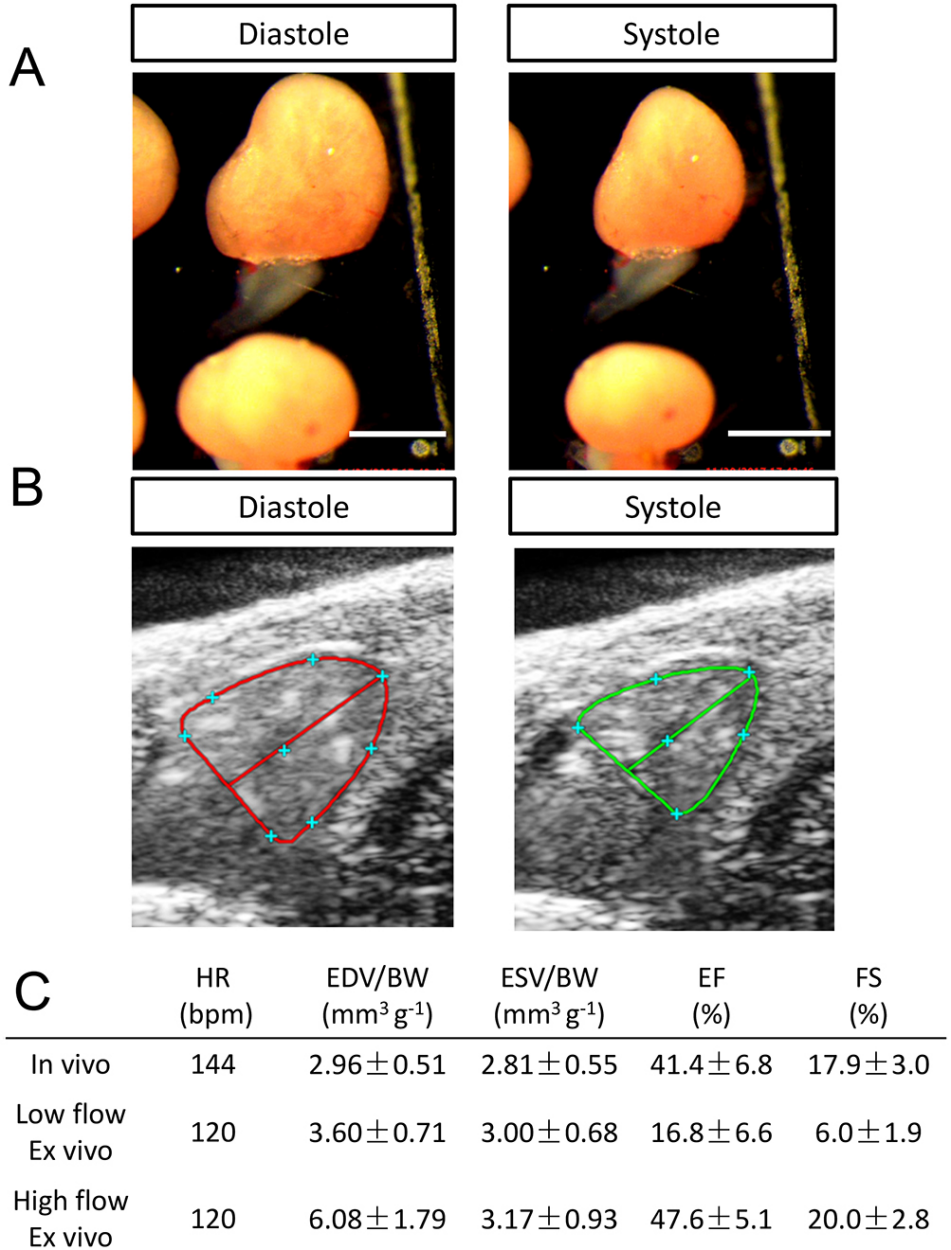
**Comparison of cardiac function measurements obtained from the *ex vivo* method and from *in vivo* HFE.** (A) Representative images of adult hearts at end-diastolic and end-systolic phases of cardiac cycle *ex vivo*. Scale bar: 1 mm. (B) Representative echocardiographic images of adult fish hearts at end-diastolic and end-systolic phases *in vivo*. (C) Parameters of the same group of 20 fish obtained from either HFE or *ex vivo* assay at low and high load. HR, heart rate; bpm, beats per minute.

To validate data generated by our new *ex vivo* system, we compared ventricular volumes and pump function with those obtained *in vivo* by HFE in a group of 20 7-month-old male TE wild-type fish. These fish were initially evaluated with HFE in Australia, shipped to the United States, and then reassessed after acclimatization with the *ex vivo* system (Movie 2). Comparable EFs were obtained when *ex vivo* experiments were conducted at a flow rate of 0.4 ml min^−1^ (high flow; *in vivo*, 41.4±6.8%; *ex vivo*, 47.6±5.1%; Fig. 5C; Fig. S3C). Results were similar for FS (Fig. S3D). However, ventricular volumes measured *ex vivo* at the 0.4 ml min^−1^ flow rate were about twice those obtained *in vivo* in the same fish (*in vivo*, EDV/BW, 2.96±0.51 mm^3^ g^−1^; *ex vivo*, 6.08±1.79 mm^3^ g^−1^; Fig. 5C).

We reasoned that these volume differences were driven by the relatively higher hemodynamic load used in the *ex vivo* perfusion system. To test this hypothesis, we evaluated chamber volumes at a flow rate of 0.05 ml min^−1^ (low flow). Under these low flow conditions, mean data for *in vivo* and *ex vivo* measurements of EDV/BW and ESV/BW were comparable (Fig. 5C; Fig. S3E,F), and significant correlations were found between the 2 methods (EDV/BW, R^2^=0.75; ESV/BW, R^2^=0.78; Fig. S3G,H). For EDV/BW, there was a correlation slope of 1 and an offset of ∼0.2. For ESV/BW, in contrast, the slope was 1.6, an indication that ESV obtained *ex vivo* might be overestimated, especially for fish with large hearts. As expected, both EF and FS were markedly lower with low flow rates (Fig. 5C). In conclusion, although these studies showed excellent overall correlations between *in vivo* and *ex vivo* assessment of EDV and ESV, the absolute volumes were not directly comparable, and we attribute these differences predominantly to loading conditions.

### Additional parameters that can be measured using the *ex vivo* method

**Fig. 6.**
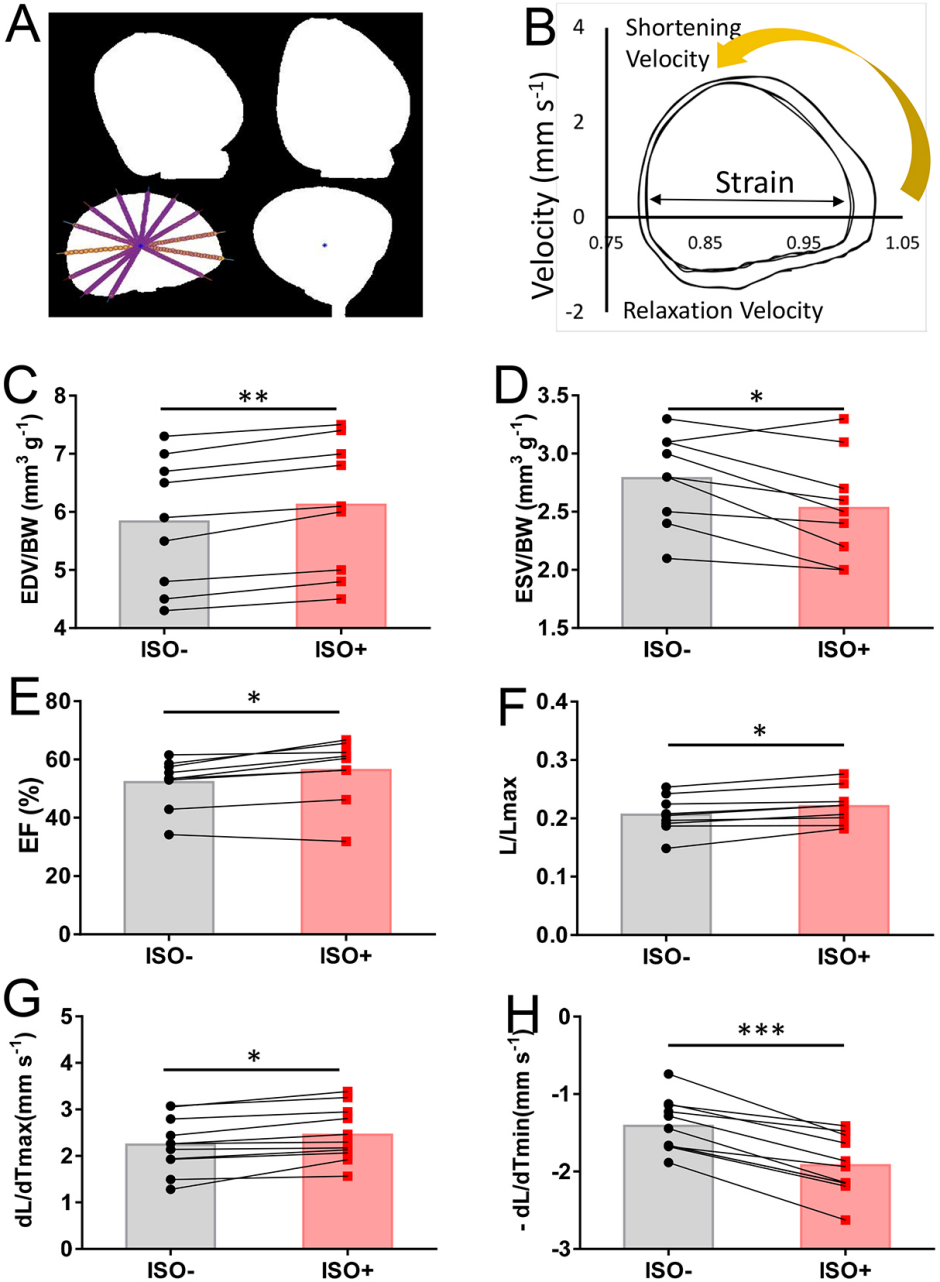
**Measurements of heart contractility and its response to β-adrenergic stimulation.** (A) The measurement of radial deformation in transverse cardiac plane using MATLAB. Color images are converted to black and white images. Radii of ventricle, set by hand, are shown in color. (B) Representative strain-velocity loops before (smaller) and after (bigger) isoproterenol injection (10^−7^ mol; 0.2 ml min^–1^). (C-E) Lusitropic and inotropic effects of bolus of isoproterenol (ISO) on volumes and EF (*n*=10). (F-H) Enhanced strain, accelerated shortening and relaxation of myocardium in response to isoproterenol. *P≤0.01; **P≤0.0001; ***P≤0.00001.

In addition to ventricular size and contractility measurements, we quantified the extent of deformation of the ventricular wall during the cardiac cycle (strain) using the *ex vivo* method. Assessment of myocardial strain has been proposed to be a sensitive indicator of contractile impairment, with potential clinical utility for detection of early disease and for serial monitoring of ventricular function (Potter and Marwick, 2018). Using a custom MATLAB code (matrix laboratory, MathWorks) (Supplemental Text), we were able to trace the edges of the ventricle during the cardiac cycle and automate calculation of ventricular strain and the velocity of myocardial wall deformation. Our setup detected silhouettes of the beating explanted hearts in 2 perpendicular projections, enabling strain to be determined in the longitudinal plane (long axis, from the atrioventricular ring to apex) and the transverse plane (short axis). We found that the average radial strain in the transverse view of the heart was less dependent on the direction of measurement and the most consistent strain parameter in our *ex vivo* system (Fig. 6A).

In mammalian Langendorff heart preparations, the rates of left ventricular pressure increase in early systole (rate of rise in pressure; +dP/dt_max_) and decrease in early diastole (rate of reduction in pressure; −dP/dt_min_), the established measures of ventricular contractility and relaxation, respectively. We developed a novel way of characterizing zebrafish cardiac function kinetics by plotting velocity (dL/dt) versus radial strain (L/L_max_). Plotting these cyclic functions against each other resulted in a loop, the dimensions of which reflect the shortening fraction (strain) along the *x*-axis and the kinetics of shortening/relaxation along the *y*-axis (Fig. 6B).

Next, we determined whether the *ex vivo* perfused zebrafish heart maintained its capacity to respond to β-adrenergic stress, and whether we were able to quantify this response using established contractile function measurements and strain. For this experiment, we chose a moderate cardiac load (0.2 ml min^−1^ flow rate) to detect the expected gain of function in response to β-adrenergic agonist stimulation. Within seconds after injection of a single bolus of isoproterenol (10^−7^ mol) into the perfusion port, the EDV increased (*P*=1.75×10^−5^; *n*=10; lusitropic effect), and the ESV decreased (*P*=0.0086; *n*=10; Fig. 6C,D). As a consequence, the EF significantly increased (*P*=0.0016; *n*=10; Fig. 6E), as did the radial strain (Fig. 6F). Then, we analyzed the kinetics of ventricular wall deformation (change of the ventricle length, L). Interestingly, the relaxation velocity response was detected first, with rate of relaxation (−dL/dt_min_) decreasing by ∼36% within 10 s after injection (Fig. 6H), and the shortening velocity response was achieved within ∼30 s, with rate of contraction (+dL/dt_max_) elevated by ∼9.5% (Fig. 6G). Together, these data indicated that our *ex vivo* zebrafish heart model allows measurement of not only routine size and contractility parameters but also additional parameters of contraction kinetics and strain.

### Evaluation of cardiac pump dysfunction in DIC using the *ex vivo* method

**Fig. 7.**
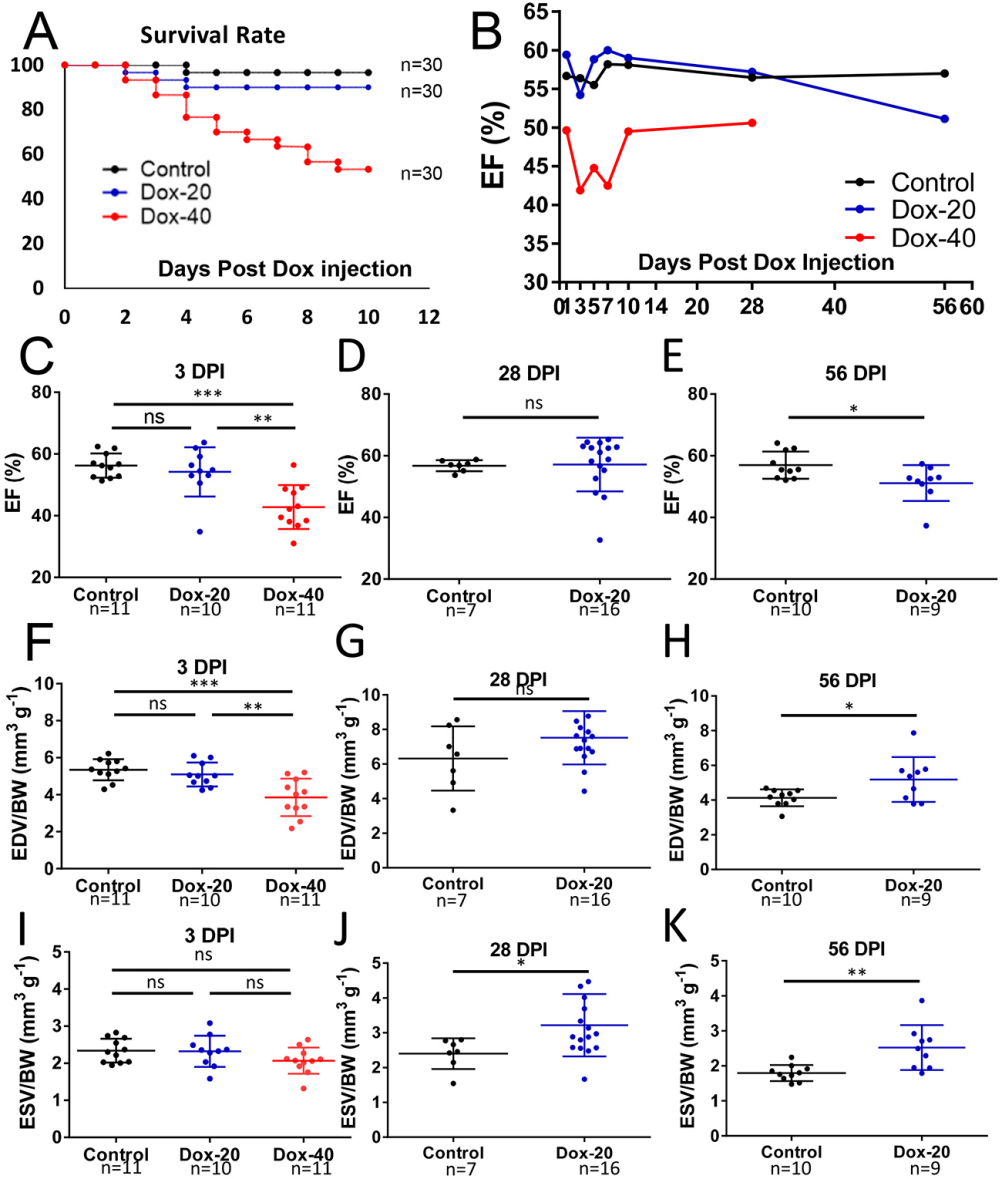
**Quantification of cardiac function *ex vivo* in DIC model.** (A) Survival of fish injected with high- (Dox-40) or low-dose (Dox-20) DOX and control group (*n*=30 for each group). (B-E) Dynamics of EF obtained from *ex vivo* experiments from fish injected with either low- or high-dose DOX and control group at 3, 7, 10, 25 and 56 dpi. Shown are either mean only (B) or detailed EF (%) at 3 dpi (C), 28 dpi (D) and 56 dpi (E). (F,G,I,J) At 3 dpi, EDV is reduced in fish injected with 40 µg g^–1^ DOX but not 20 µg g^–1^ DOX (F), but there is no difference in ESV (I). At 28 dpi, there is no difference in EDV (G), but ESV is increased (J), in fish injected with 20 µg g^–1^ DOX. (H,K) Both EDV and ESV increased at 56 dpi. Data are mean±s.d. For statistical analysis, unpaired Student's *t*-test was used for 2 groups, and one-way analysis of variance used for 3 groups. ns, nonsignificant; *P≤0.05; **P≤0.005; ***P≤0.0005.

We recently established a DIC model in adult zebrafish that manifests histological and molecular features of cardiomyopathy, including myofibrillar disarray, apoptosis and activation of fetal gene transcription (Ding et al., 2016; Packard et al., 2017). In previous work, we found reduced cardiac pump function in DOX-treated fish using video analysis of fluorescent hearts in the adult transparent *Casper; Tg(cmlc2:DsRed)* line (Ding et al., 2011; Hoage et al., 2012). We wanted to determine whether the newly developed *ex vivo* assay was sufficiently sensitive to detect cardiac dysfunction in wild-type fish exposed to an equivalent DOX dose (20 µg g^–1^) (Fig. 7A,B). Compared with control fish, there were no differences in EF at 3 days postinjection (dpi) (54.2±8.0%), 7 dpi (60.0±1.6%) and 28 dpi (57.2±8.7%), but significant reductions were apparent at 56 dpi (51.1±5.8%) (Fig. 7C,E). Consistent with this, EDV/BW (5.19±1.29% vs 4.13±0.49%) and ESV/BW (2.65±0.73% vs 1.79±0.23%) were increased at 56 dpi (Fig. 7H,K), similar to the late-onset dilated cardiomyopathy found in humans after chemotherapy. When the dose of DOX was increased to 40 µg g^–1^, we noted a significant reduction in EF during the first week postinjection (41.88±7.13%), which later recovered at 10 dpi (49.49±5.04%), a reflection of an acute toxic effect of DOX to the heart (Fig. 7B). This reduced EF at 3 dpi (41.88±7.13%) was accompanied by reduced EDV/BW (3.86±1.01% vs 5.35±0.57%) and unchanged ESV/BW (2.07±0.35% vs 2.34±0.32%) (Fig. 7F,I).

Taken together, using our *ex vivo* assay, we found that high doses of DOX caused acute early cardiotoxicity, whereas lower doses led to a later-onset cardiomyopathy ∼8 weeks postinjection.

## DISCUSSION

### An *ex vivo* assay to quantify cardiac pump function in adult zebrafish

We have described a novel method for quantification of cardiac pump function in explanted adult zebrafish hearts. This method was based on our previous studies in which we used isolated single zebrafish ventricular cardiomyocytes (Bovo et al., 2013; Dvornikov et al., 2014). We used fish Tyrode buffer to provide the isolated ventricle with sufficient glucose and pyruvate energy sources and maintained a constant heart rate on electrical field stimulation. Schematics of the whole-heart perfusion system were adapted from the perfusion systems described previously (Llach et al., 2011; Zhang et al., 2011). Unlike Langendorff preparations of mammalian hearts that rely on coronary artery perfusion, the small dimensions of the zebrafish heart allowed oxygen and nutrient diffusion from the luminal side. Because extensive excision of the atrium was required for proper heart fixation in a field of view, our assessment was focused primarily on ventricular pump function.

We evaluated the impact of experimental conditions on the function of explanted ventricles, including perfusion time, flow rate (load), [Ca^2+^]_o_ in the perfusion buffer and pacing frequency. We established standard measurements of ventricular size and contractility and developed advanced methods to measure strain and velocity of contraction/relaxation via ventricular wall deformation analysis. Although pressure-volume loops derived from conductivity catheters inside the left ventricle have been used as a standard for *in vivo* cardiac function assessment in rodents (Gut et al., 2017), this is not possible in zebrafish hearts because of their small size. However, in the described *ex vivo* system, we demonstrated the feasibility of generating velocity-strain loops as an alternative approach. Plotting the rate of deformation against the deformation might help in the assessment of mechanical work performed by the isolated zebrafish ventricle.

To assess the sensitivity of our novel *ex vivo* technique to detect perturbation of cardiac contraction, we evaluated the effects of hypercontractile and hypocontractile states induced by β-adrenergic stimulation or DOX treatment, respectively. Indeed, after isoproterenol treatment, rapid changes in ventricular size and contractility were detected, opening new opportunities for utilizing zebrafish *ex vivo* heart preparations for drug studies. High-dose DOX exposure resulted in acute toxic cardiomyopathy with reduced ventricular size and contractility during the first week postinjection, whereas low-dose DOX treatments caused later-onset dilated cardiomyopathy with ventricular dilatation and contractile dysfunction within 8 weeks postinjection. These 2 distinct responses to DOX exposure are consistent with our previous observation, in which mammalian target of rapamycin signaling pathways are differentially activated during early and late DOX cardiotoxicity (Ding et al., 2011, 2012), and argue in favor of a stage-dependent therapeutic strategy. This *ex vivo* heart model system is anticipated to facilitate future mechanistic studies and therapeutic development in zebrafish models of cardiomyopathy.

### Zebrafish versus mammalian Langendorff models

In mammalian Langendorff models, various perfusion agents and methods have been used, ranging from simple retrograde saline perfusion (Langendorff, 1898) to working-heart models operating with blood-containing buffers. In typical Langendorff preparations, the heart is connected to the perfusion system by way of the aorta, with retrograde perfusion of the coronary arteries and ventricles (Langendorff, 1898). The left ventricular cavity is occupied by a latex balloon. This not only enables left ventricular pressure to be measured but also maintains positive end-diastolic pressure and constant wall stretch and provides isovolumic conditions for cardiac contractions, mimicking physiological conditions.

According to the Frank–Starling law of the heart, there is a positive relationship between ventricular force development and sarcomere length, and hence ventricular pressure is a key determinant of contractile function. In contrast to mammalian Langendorff models, ventricular pressure in the zebrafish *ex vivo* model is maintained by flow rather than by balloon stretch. The constant anterograde flow through the isolated ventricle generates a constant positive intraventricular pressure with minimal afterload, aside from OFT resistance. Without a catheter (or with damaged OFT), the lack of pressure results in minimal heart contractions (Movie 3), and thus quantification of cardiac functions is inaccurate.

### Comparison of *ex vivo* and *in vivo* methods for cardiac function assessment

We used our *ex vivo* method to determine heart size and function in male and female wild-type fish at 3, 6, 9 and 12 months of age. Similar to recent HFE findings (Wang et al., 2017), we found that *ex vivo*-determined EF and FS were stable over time and sex independent. Furthermore, as found with HFE, apparent effects of age and sex on EDV and ESV were abolished by normalization to body size and weight. The overall high correlations between *in vivo* and *ex vivo* data support the use of our *ex vivo* system as an alternative method for cardiac size and function assessment, particularly when HFE equipment is not accessible or serial studies are not required.

We found that EDV initially increased with increasing flow rate up to a maximum of 0.6-0.8 ml min^–1^, representing the end of elasticity reserve (data not shown). Higher flow rates are unlikely to be tolerated and might have deleterious effects on myocardial structure and function. Going up to maximal load and then back to a flow rate of 0.2 ml min^−1^ can help to detect not only contractility rundown but also parameters of myocardium elasticity. Although high loading (up to a maximum of 0.6-0.8 ml min^−1^) might represent a relatively hyperdynamic state, these conditions were optimal for assessment of cardiac contractility.

These supraphysiologic operating conditions of the *ex vivo* system need to be taken into account when comparing absolute values of cardiac function indices with those obtained by HFE. In particular, ventricular size was up to 2-fold larger than that measured by HFE. This finding was not surprising, given that perfusion rate was also 2-fold larger than physiologic cardiac output *in vivo*. However, variability in defining the inner border of the ventricular compact layer in HFE and potential angle errors in the imaging plane could contribute to discrepancies between the *ex vivo-* and *in vivo*-derived data. Nonetheless, comparable EF/FS values and the good correlation of EDV/ESV between the 2 methods (Fig. S3G,H) underscore the validity and utility of the *ex vivo* system. Of note, most experiments are designed to compare relative changes in cardiac function indices in the experimental versus control groups, with absolute values of each index less relevant. As exemplified by our study of the DIC model, the *ex vivo* assay was sufficiently sensitive to detect distinct cardiac dysfunction characteristic of different phases of DIC pathogenesis.

### Limitations and advantages of the *ex vivo* method

Our new *ex vivo* approach has several limitations. First, hemodynamic and other functional conditions are obviously different from the *in vivo* situation, and the invasive nature of the method means that fish need to be sacrificed; thus, paired serial assessments in single animals are not possible. This disadvantage is partially offset by the fact that, as a result of the economics of fish husbandry, large numbers of adult zebrafish can be raised and studied. Second, we used the epicardial ventricular border to calculate ventricular volumes but not the inner border of the ventricular compact layer, which can be visualized by HFE (Wang et al., 2017). This approach might have contributed to the differences between *ex vivo* and *in vivo* volume measurements. Light-sheet microscopy is an alternative method to quantify ventricular lumen/volume (Ding et al., 2017; Packard et al., 2017; Vedula et al., 2017); however, this approach is limited to postmortem fluorescent noncontracting hearts.

Our data suggest that *ex vivo* isolated heart preparations do have many advantages over *in vivo* studies. Confounding factors that can contribute to variability in heart function measurements during echocardiography, such as neurohumoral influences, the degree of anesthesia and associated changes in heart rates, can be effectively eliminated. The ventricular epicardial border is clearly delineated *ex vivo,* whereas ascertainment of myocardial borders in HFE requires a considerable extent of operator expertise. The most important advantage of our *ex vivo* method is the ability and scope for experimental manipulation, which opens new research opportunities. Variables such as ventricular preload, the level of activator Ca^2+^ in the perfusion buffer, and the frequency of stimulation can be easily adjusted to experimental needs. Dynamic changes in contractile function on different loads, which can be conveniently plotted using the *ex vivo* method, might facilitate the detection of latent myocardial defects that are not apparent under physiologic conditions. Furthermore, pharmacologic agents can be readily applied and acute cardiac responses directly monitored with the *ex vivo* method. Because heart function in isolated fish ventricles is stable for more than 20 min, multiple pharmacologic manipulations are feasible. Our perfusion system can also be easily extended to optical mapping experiments, whereby excitation and Ca^2+^ wave propagation can be studied through the myocardium (Werdich et al., 2012). Together with HFE, this new method is anticipated to further promote the use of adult zebrafish as a vertebrate model for studying human cardiac diseases.

## MATERIALS AND METHODS

### Fish preparation

Zebrafish were handled with care, according to the guidelines of the Mayo Clinic Institutional Animal Care and Use Committee (protocol number A00002783-17). Adult wild-type zebrafish of *WIK* and *NHGRI-1* (TAB) background were used in this study (LaFave et al., 2014). To ensure consistent growth, the density of fish was 30 per 2.8-l medium tank at 28°C.

### Cardiac perfusion system

Our perfusion system consists of a pump, an isolated MyoPacer stimulator (IonOptix), a perfusion plate, and a stereo microscope (Fig. 1A). The peristaltic pump EP-1 Econo Pump (Bio-Rad) is used to control the flow of the perfusion solution into the isolated zebrafish heart. An explanted ventricle is sutured onto the tip of a 34G catheter placed onto the perfusion plate, and the perfusion plate is set onto a custom-made black Sylgard (Sylgard 184; Dow Chemical Company)-coated Petri dish (Fig. 1B). We used adhesive removable putty to make a pool to control the volume of the solution and to place the fulcrum of the glass. The entire perfusion setup was placed under a M165C stereo microscope (Leica Microsystems) (Fig. 1C). Two platinum electrodes from a stimulator were placed into the solution, on each side of the heart. To obtain a head-on view and a lateral view at the same time, we placed a 5-mm aluminum 45° right-angle mirror (Thorlabs) in front of the heart. To prevent image distortion resulting from a spherical droplet, a thick coverslip was placed on top of the pool to keep the surface of the solution flat.

The fish Tyrode solution was modified according to the method of Llach et al. (2011), and contains 132 mM NaCl, 2.5 mM KCl, 4 mM NaHCO_3_, 0.33 mM NaH_2_PO_4_, 1 mM CaCl_2_, 1.6 mM MgCl_2_, 10 mM HEPES, 5 mM glucose and 5 mM sodium pyruvate. pH was adjusted to 7.5 with NaOH. All experiments were performed at room temperature.

### Surgical procedures

Fish were anesthetized by immersion in ice-cold E3 water containing 0.16 mg ml^–1^ tricaine methanesulfonate (Western Chemical) for 2-5 min before being measured and weighed. The fish heart was dissected and transferred to a perfusion plate containing perfusion saline buffer. The thin-walled atrium was torn open using forceps, the tip of a 34G catheter inserted through the atrioventricular valve into the ventricle to allow anterograde perfusion of the heart, and the opened atrial tissue sutured onto the catheter using 10-0 Prolene (USSC) (Fig. 1C). The ventricle was always positioned with the long axis perpendicular to the plate, OFT facing down, surrounded by solution without touching the plate, to ensure consistent positioning relative to the mirror. To prevent damage during surgery, great care was taken to avoid touching the ventricle or OFT with the forceps. Our system was designed to allow mounting of 2 explanted hearts simultaneously using 2 catheters connected to a 2-channel pump. All surgical procedures were performed in the presence of 0.05 ml min^−1^ flow rate, which facilitates operation. The entire procedure from the onset of fish anesthesia to the first heart recording was routinely achieved within 5-10 min.

### Cardiac pacing, perfusion rates, image acquisition and compound treatment

Heart beating was maintained by electrical field stimulation generated between 2 platinum electrodes placed in the saline buffer on either side of the heart. The voltage of the stimulation impulse was set at 10-20% above threshold (∼15 V) and the duration was set at 10 ms. The initial flow rate of the perfusion solution was set at 0.05 mg ml^–1^. After 1 min, when near steady-state conditions were reached, high-speed videos of the beating hearts were recorded for 5-6 s (∼10 cardiac cycles), as described below. We then adjusted the perfusion flow rate to 0.1, 0.2, 0.4, 0.6, 0.8 or 1.0 mg ml^–1^ (depending on BW) and documented corresponding videos at each step. As a control, we always returned the flow rate to a lower flow rate, such as 0.4 ml min^−1^, as a final step, waited for 3 min to reach steady state, and recorded a video again. Pump functional indices could be compared with those from an earlier time point with the 0.4 ml min^−1^ flow rate, which shall determine whether there is pump function rundown or heart rupture during the experimentation.

For drug treatment, a bolus of 0.2 ml isoprenaline hydrochloride (Sigma-Aldrich; 10^−7^ mol per heart) was injected into each perfusion channel by syringe through an injection port. Within a minute of injection, a series of 3 video files was documented. Measurements from the 3 videos were averaged for data analysis.

### Image acquisition and ventricular volume calculation

A 14-MP color CMOS high-speed camera (Amscope, MU1403) was used to record movies (Leica-MPEG compression) at 66 frames s^–1^ with an exposure time of 10 ms. A minimum of 10 cardiac cycles was recorded for each video using Amscope v3.3.7934 acquisition software (Amscope). Ventricular parameters were measured from at least 2 representative cardiac cycles using ImageJ software (National Institutes of Health).

Ventricular chamber dimensions were obtained from 2 projections: direct projection [transverse, or short axis (SAX)] and mirror projection [longitudinal, or long axis (LAX)]. Systolic and diastolic areas (A_s_ and A_d_, respectively) can be obtained by measuring the area of ventricle using the polygon selection tool from the desired projection at end-systole and end-diastole, respectively. In LAX, the ventricular lengths (L_s_ and L_d_) were measured as the distance from the base to the apex at end-systole and end-diastole, respectively.

To calculate ventricular volumes from 2 perpendicular projections of the heart, we used the area-length formula for a half-ellipsoid shape (Lang et al., 2005; Tortoledo et al., 1983; Wahr et al., 1983), with *V*=(2/3)*AL*, where A is the area of the base of the ventricle in SAX and L is the length of the ventricle in LAX. However, for different purposes, different formulae can be used; for instance, for the echocardiography single-plane (LAX) images, a single-plane formula might be preferable. To standardize ventricular volumes in fish of different sizes, we normalized them to BW, BL (defined as maximal length from snout to base of tail), BMI [calculated as BMI=weight/(length)^2^] and BSA (calculated using the formula BSA=8.46×weight 0.66), as described previously (Wang et al., 2017).

Cardiac contractility was quantified using EF [EF=(EDV−ESV)/EDV] or FS [FS=(L_d_−L_s_)/L_d_], where we recommend to average FS from the LAX and SAX planes, if possible. (Note that FS is calculated from LAX.)

Average radial strain (L/L_max_), maximal shortening velocity (+dL/dt_max_) and maximal relaxation velocity (−dL/dt_min_) were calculated using MATLAB code applied to SAX plane. Briefly, images of 2 hearts in 2 projections were converted to binary images. After application of filters (by size and threshold), only ventricle projections remained on the images. Then, deformation was calculated as a change in length of the average radius from the edges of the ventricle to a center of mass, which was recalculated every frame using standard MATLAB function. The first time derivative of deformation (L), measured frame to frame at 66 frames s^–1^, equals the strain velocity (dL/dt). Maximal velocities of shortening/relaxation were calculated as extremums of the function. The original MATLAB code can be found in the Supplemental Text.

### DOX injection

Doxorubicin hydrochloride (DOX; Sigma), 20 µg g^–1^ BW or 40 µg g^–1^ BW, was injected intraperitoneally (i.p.) into anesthetized 6-month-old wild-type zebrafish to induce cardiomyopathy using a 28 G needle, as previously described (Ding et al., 2011). Cardiac contractility was evaluated in fish sacrificed at 1, 3, 5, 7, 10, 28 and 56 dpi (7 time points), with *n*=6 to 18 for each time point. We repeated experiments in 3 different batches of injected fish. Each batch had 3 groups and each group contained 30 fish (in total, 90 fish). For each time point, we combined 3 batches of fish.

### Statistical analysis

All statistical analyses were performed with JMP software v10 (SAS Institute). Significant differences for normally distributed continuous variables between groups were determined with unpaired Student's *t*-tests, and repeated measurements within groups were analyzed with paired Student's *t*-tests. Differences between measurements at multiple time points were determined by repeated measures analysis of variance. Factor analysis was performed with multivariate analysis of variance. *P*<0.05 was considered significant. Data are presented as mean±s.d.

## Supplementary Material

Supplementary information
