# Supplementary material for: A Langendorff-like system to quantify cardiac pump function in adult zebrafish
Source: Dis Model Mech. 2018 Sep 10;11(9):dmm034819. doi: 10.1242/dmm.034819 (PMC6177000; doi:10.1242/dmm.034819)
Supplement: Supplementary information [file dmm-11-034819-s1.pdf]

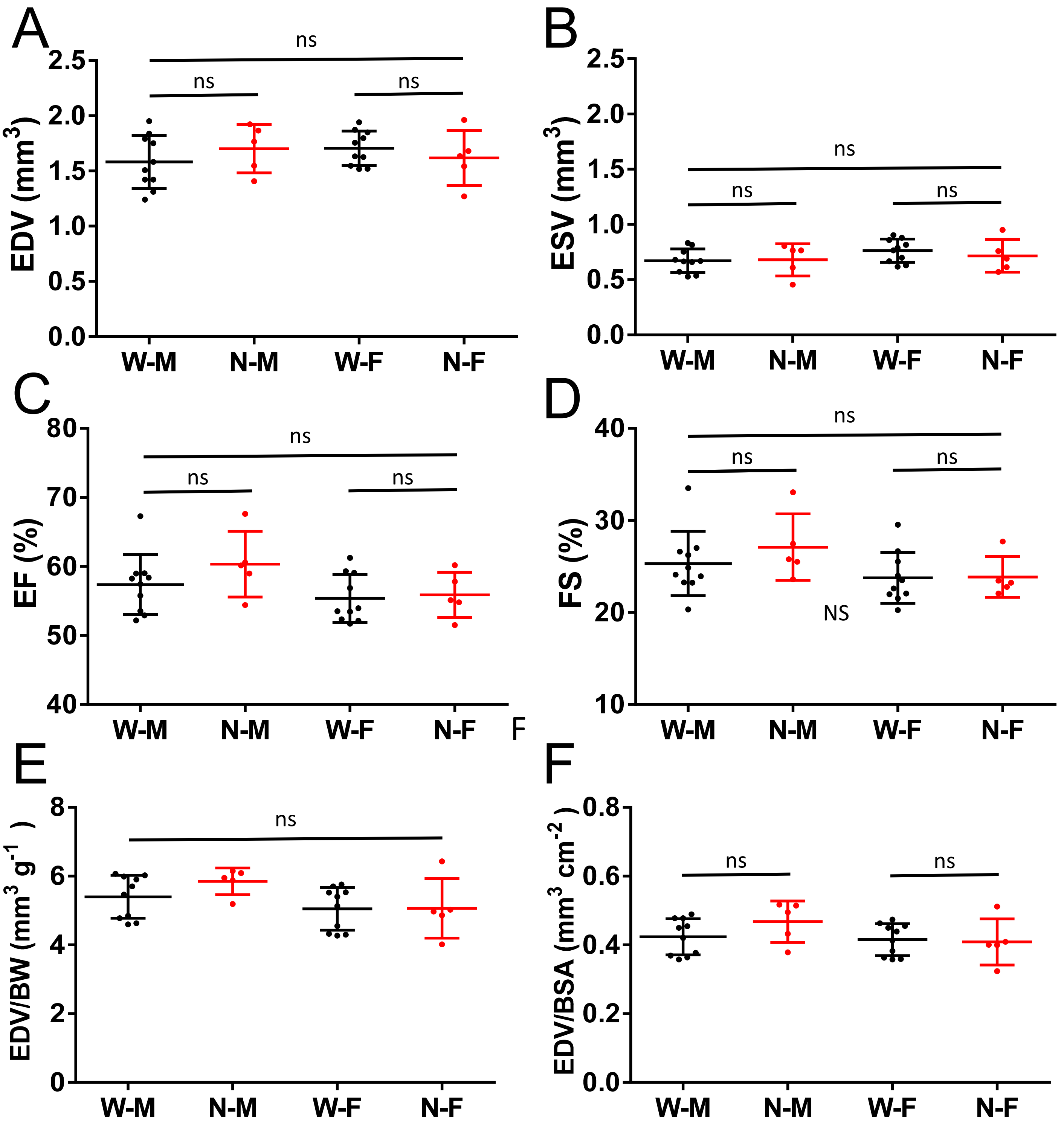

Fig. S1. Comparison of Baseline Cardiac Functions Between 2 Different Wild-Type Lines. The measurements were obtained from 6-month-old fish from 2 wild-type strains, WIK (W) (10 fish per sex) and NHGRI-1 (N) (5 fish per sex). Shown are quantifications of end-diastolic volume (EDV) (A), end-systolic volume (ESV) (B), ejection fraction (EF%) (C), fractional shortening (FS) (D), EDV/BW (body weight) (E), and EDV/BSA (body surface area) (F). Data are mean (SD). Unpaired *t* test used for 2 groups, and one-way analysis of variance was used for 3 or more groups. ns indicates nonsignificant.

Disease Models & Mechanisms • Supplementary information

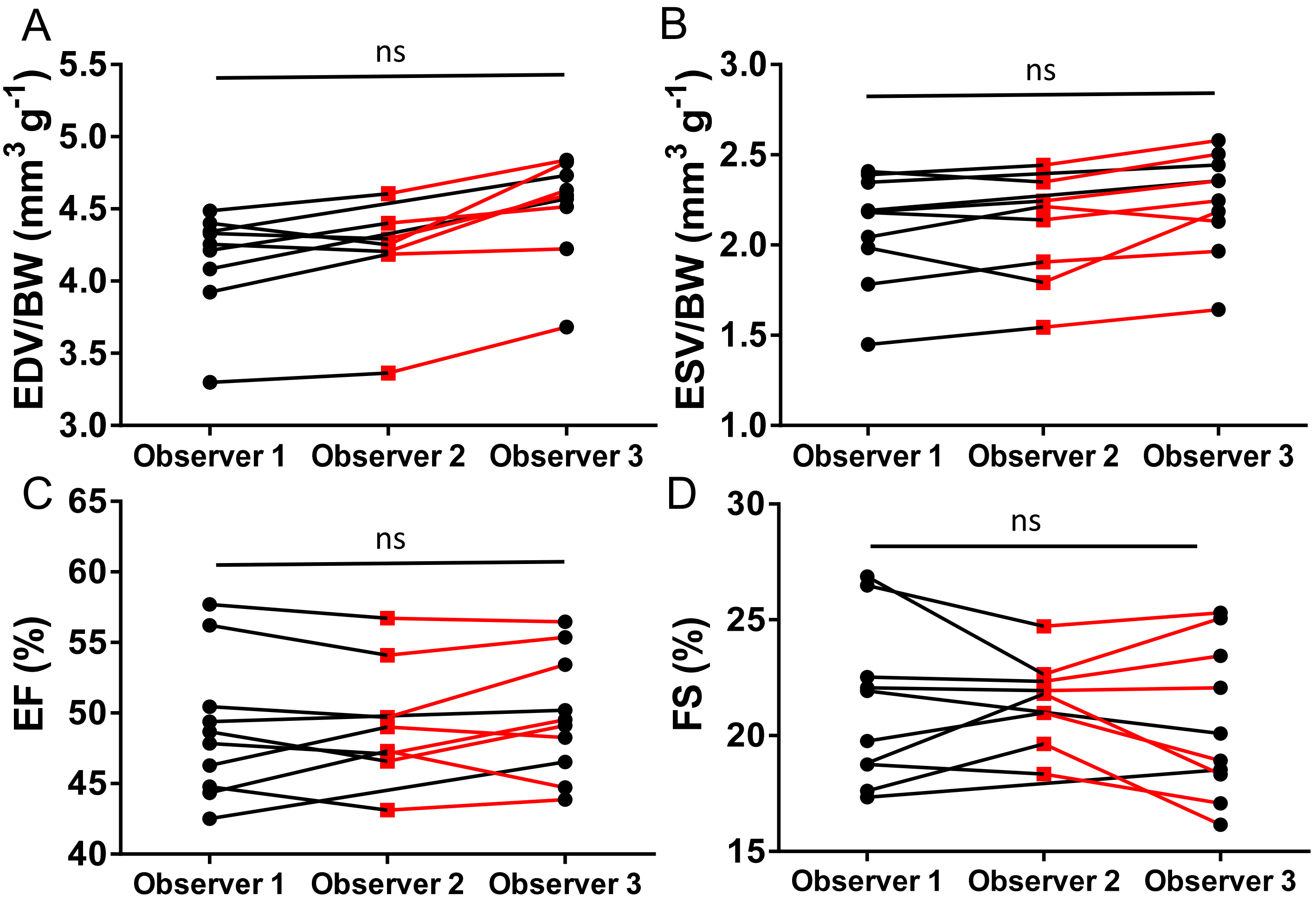

Fig.S2. Interobserver Variability and Repeatability. Observers 1 through 3 were blind when analyzing 10 videos obtained from ex vivo assay. Shown are quantifications of end-diastolic volume (EDV) (A), end-systolic volume (ESV) (B), ejection fraction (EF%) (C), and fractional shortening (FS) (D). Data are mean (SD). Analysis of variance for repeated measures was used for these 3 groups. ns indicates nonsignificant.

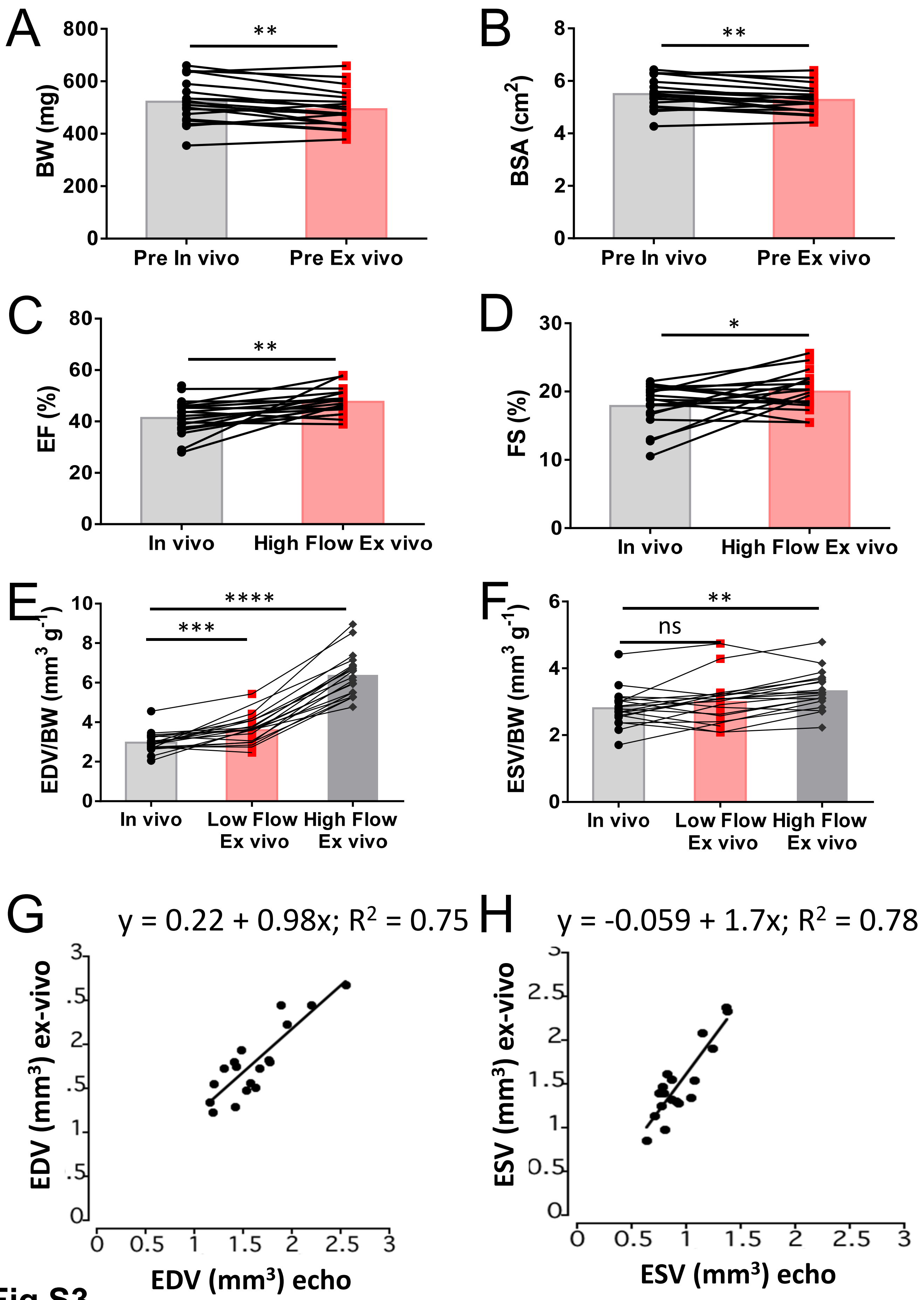

Fig.S3.

Disease Models & Mechanisms • Supplementary information

Fig.S3. Comparisons Between In Vivo High-Frequency Echocardiography (HFE) and Ex Vivo Measurements. A through D, Both ejection fraction (EF) and fractional shortening (FS) obtained by the ex vivo method at high load are higher than those obtained using HFE. E and F, End-diastolic volume (EDV) and end-systolic volume (ESV) obtained with the ex vivo method at low load are more similar to those obtained with HFE. G, Linear regression analysis shows significant correlation of EDV obtained using the 2 methods. H, Linear regression analysis shows significant correlation of ESV obtained using the 2 methods. Data are mean (SD). Paired *t* test was used. ns indicates nonsignificant; **P*≤.05; ***P*≤.005; ****P*≤.0005; *****P*≤.0001.

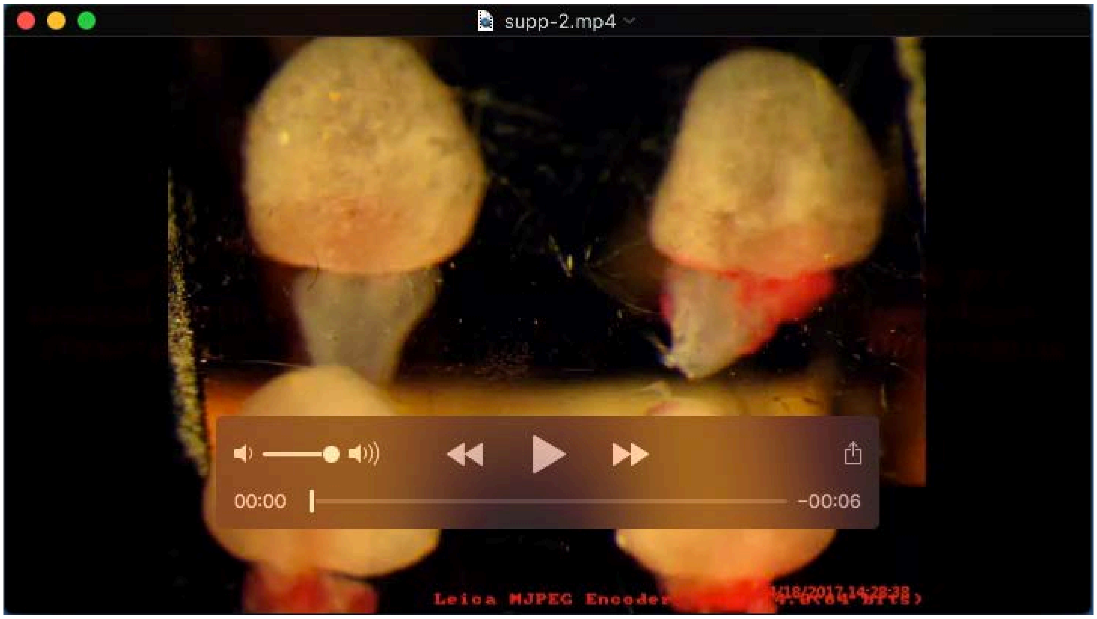

Movie 1. A representative movie of adult hearts with (left) or without (right) doxorubicin injection.

Disease Models & Mechanisms • Supplementary information

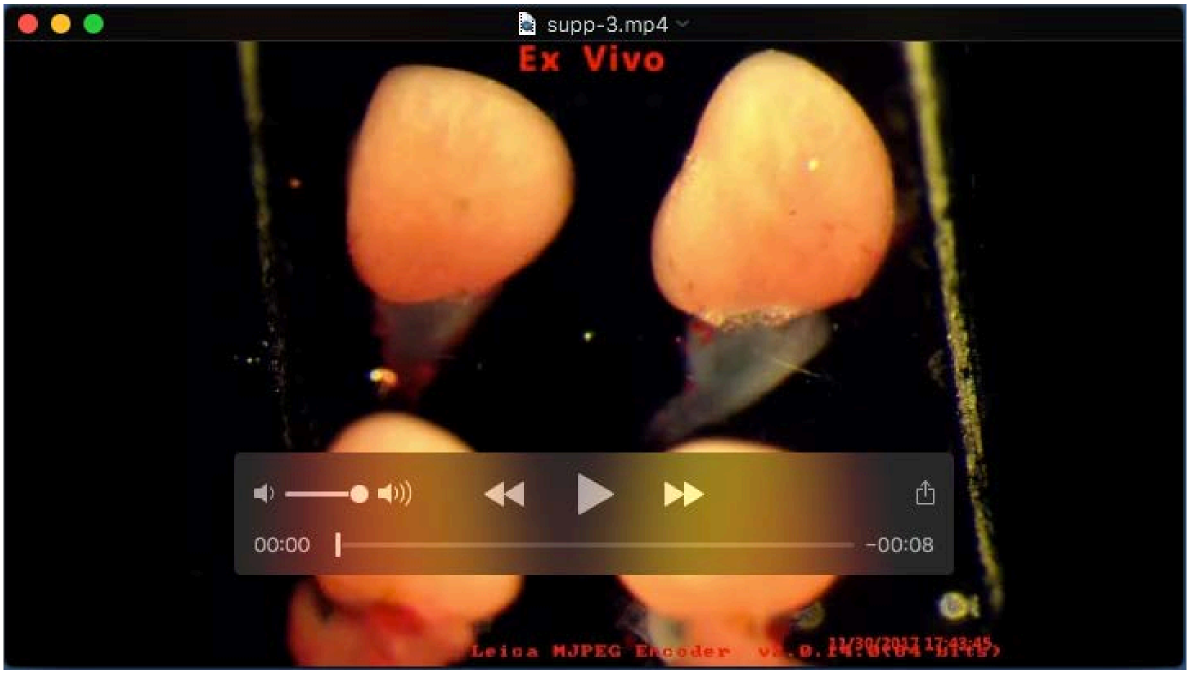

Movie 2. A video clip shows 2 beating hearts using ex vivo system, which is followed by another video clip that shows the same heart on the right side by echocardiography.

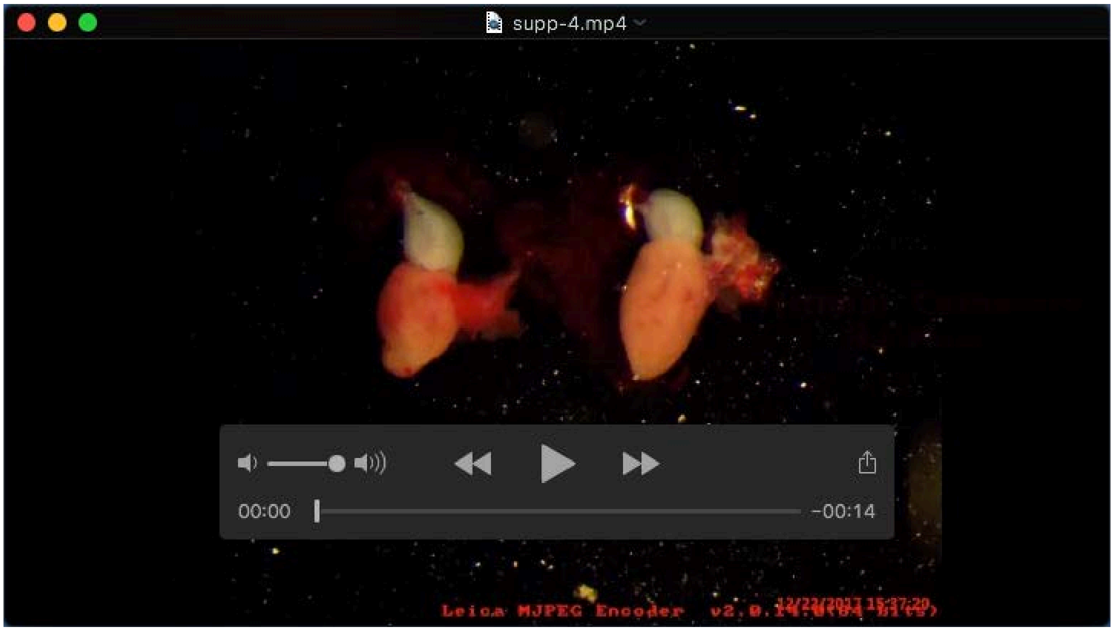

Movie 3. The same hearts without catheters, with catheters but no flow, and with catheters and flow.

Supplemental Text. Matlab code to analyze radial strain and velocity.

```
% Script to open avi files of beating hearts
% 4 images of heart are allowed;
% (c)Alexey V Dvornikov 2017 version 2.03
% one cutting line (c) and radial strain measurement
% slide frame filtering of first derivative before extremum detection
% B&W image
v = VideoReader('18ja29_3_2bbbb.avi');%reading file;

nframes = v.NumberofFrames; duration = v.Duration; nframes / duration %show fps
pixelsize = 200; %(200 or 160) if zoom is 2x (reversed size of pixel (pixels/unit))
nframes1 = 20; %(20) defZult number of frames to detect diastole
nframes2 = 100; %(100) finite number of frames for the second cycle
thres = 0.15; %(0.15) threshold for making binary image
size = 20000; %(15000) size of min areas (big hearts - 22000)
disk = 5; %(5) size of disk objects
%%%%%zeroing%%%%%
Dvector1 = [];Dvector2 = [];Dvector = []; Dvector3 = [];
Avector = []; Arows = []; A2 = []; Dvector4 = []; Dvector5 = 0;
Dvector6 = []; Dvector7 = []; Dvector8 = []; Dvector4f = [];

for l=1:nframes1 %cycle to find a diastole
   singleFrame = read(v, l);
   I = im2bw(singleFrame, thres);
   sedisk = strel('disk', disk); %size of disk object
   I1 = imopen(I, sedisk);
   I2 = bwareaopen(I1, size); %remove small objects
   stats = regionprops(I2,'Area');
   A1 = [stats.Area];
   Avector=[Avector;A1(1:4)];%put 4 of these areas in array
   Arows=[Arows;sum(Avector(l,:)),l];%sums of these 4 areas in a column
end
%Diastoleframe
A = max(Arows);
B = find(Arows ==A(1)); %find a maximum index ==> frame
singleFrame = read(v, B);
BW = rgb2gray(singleFrame);
imshow(BW)

%stop & line dialogue
c1 = imline(gca); % horizontal cutting line c1
%c2 = imline(gca); % vertical cutting line c2
%id = addNewPositionCallback(g,@(pos) title(mat2str(pos,3)));
m = impoly(gca); %measurement poly

posg = getPosition(c1);
%posj = getPosition(c2);
pos = getPosition(m);
npos = numel(pos)/2;%number of points in polygon

xg1 = posg(1,1); yg1 = posg(1,2); xg2 = posg(2,1); yg2 = posg(2,2);%coordinates of c1.line
%xj1 = posj(1,1); yj1 = posj(1,2); xj2 = posj(2,1); yj2 = posj(2,2);%coordinates of c2.line
xxg = linspace(xg1,xg2,abs(xg2-xg1));%make array of new XX.c1 with the step of pixel
%xxj = linspace(xj1,xj2,abs(xj2-xj1));%make array of new XX.c2 with the step of pixel
%yyj = linspace(yj1,yj2,abs(yj2-yj1));%make array of new YY.c2 with the step of pixel

yyg = spline([posg(:,1)],[posg(:,2)],xxg);%interpolate new YY.c1 to these points
%yyj = spline([posj(:,1)],[posj(:,2)],xxj);%interpolate new YY.c2 to these points
%xxj = spline([posj(:,2)],[posj(:,1)],yyj);%interpolate new XX.c2 to these points
%plot(xx,yy,'b*')
xxg = int16(xxg); yyg = int16(yyg);
%xxj = int16(xxj); yyj = int16(yyj);

stats = regionprops(I2,'centroid');%find centroids
   centroids=cat(1,stats.Centroid);
   ncentr = numel(centroids)/2;%number of areas
   ZA=[]; ZB = []; z=1;

   for s = 1:ncentr %find centroid near polygon
      ZA = [(sum(pos(:,1))/(numel(pos)/2)),(sum(pos(:,2))/(numel(pos)/2))] - centroids(s,:);
      ZB = (sum(abs(ZA))/2)^2;
      if ZB < 5000; %(3000)
         z=s;
      end
      A2 = [A2;ZB];
   end
area1 = 0;
Avector = [];
Arows = [];cent = [];centroids = [];
%%%%%%%%MAIN LOOP%%%%%%%%%%%%%%%%%%%%%%%%%%%%%
for k = B : B+nframes2 %loop starts from diastole to +nframes2
%for k = 1 : nframes %loop starts from beginning to +nframes
   singleFrame = read(v, k);%read frame, convert to grayscale; this is unit8 array
   I1 = im2bw(singleFrame, thres); %make a binary image;set a threshold (0.1)
   sedisk = strel('disk', disk); %size of disk object (5)
   I2 = imopen(I1, sedisk);

   for p=1:numel(xxg)%draw a thick black cutting line c1
   I2(yyg(p),xxg(p)) = 0;
   I2(yyg(p)+1,xxg(p)) = 0;
   I2(yyg(p)-1,xxg(p)) = 0;
   I2(yyg(p),xxg(p)+1) = 0;
   end
```

```
%    for q=1:numel(xxj)%draw a thick black cutting line c2
%    I2(yyj(q),xxj(q)) = 0;
%    I2(yyj(q)+1,xxj(q)) = 0;
%    I2(yyj(q)-1,xxj(q)) = 0;
%    I2(yyj(q),xxj(q)+1) = 0;
%    end

    I2 = bwareaopen(I2, size); %remove all small objects below 'size' (20000)

    stats = regionprops(I2,'centroid');%find centroids every frame
    centroids=cat(1,stats.Centroid);

    imshow(I2) %%%%%%%%%%%%% binary image OR
    %imshow(singleFrame)%%%%%% color image

    hold on

    xx2 = 0; yy2 = 0; xxn2 = 0; yyn2 = 0;
    for b = 1:npos
        x1 = pos(b,1); y1 = pos(b,2); x2 = centroids(z,1); y2 = centroids(z,2);

        xx = linspace(x1,x2,abs(x2-x1));
        yy = spline([centroids(z,1),pos(b,1)],[centroids(z,2),pos(b,2)],xx);

        yyn = linspace(y1,y2,abs(y2-y1));
        xxn = spline([centroids(z,2),pos(b,2)],[centroids(z,1),pos(b,1)],yyn);

        xx = int16(xx); yy = int16(yy);
        xxn = int16(xxn); yyn = int16(yyn);

        catX = 0;
        for m=1:numel(xx)%calculate how many white pixels (in X:catX) in the line m1
        xx1 = xx(m);
        yy1 = yy(m);
            if I2(yy1,xx1) == 1;
            xx2 = [xx2;xx1];
            yy2 = [yy2;yy1];
            catX = catX+1;
            end
        end

        catY = 0;
        for r=1:numel(yyn)%calculate how many white pixels (in Y:catY) in the line m1
        xxn1 = xxn(r);
        yyn1 = yyn(r);
            if I2(yyn1,xxn1) == 1;
            xxn2 = [xxn2;xxn1];
            yyn2 = [yyn2;yyn1];
            catY = catY+1;
            end
        end

        %calculate hypothenuses
        D = sqrt((catX^2)+(catY^2));
        Dvector = [Dvector,D/pixelsize];
        plot([centroids(z,1),pos(b,1)],[centroids(z,2),pos(b,2)])
        scatter(xx2,yy2)
        scatter(xxn2,yyn2)
    end
    plot(centroids(1,1), centroids(1,2),'b*');%show centroid #1
    plot(centroids(3,1), centroids(3,2),'b*');%show centroid #3
    hold off

    Dvector1 = [Dvector1;Dvector];
    Dvector = [];

end

Dvector2 = diff(Dvector1);
Dvector3 = mean(Dvector1,2);%average strain or displacement
Dvector4 = mean(Dvector2,2);%average velocity

for u=1:(numel(Dvector4)-4)%sliding frame ave filter (5) for derivative signal
Dvector4f = [Dvector4f;(Dvector4(u) + Dvector4(u+1)+ Dvector4(u+2) + Dvector4(u+3) + Dvector4(u+4))/3];
end
D1 = 0; D2 = 0;
%Dvector5 = round(Dvector4, 2)
Dvector5 = findpeaks(Dvector3); %maxima
Dvector6 = 1-findpeaks(1-Dvector3);%minimums
Dvector7 = findpeaks(Dvector4f);%relaxation velocity -dR/dt
D1 = find(Dvector7>0.009);
Dvector7 = Dvector7(D1);
Dvector8 = 1-findpeaks(1-Dvector4f);%contraction velocity +dR/dt
D2 = find(Dvector8<0);
Dvector8 = Dvector8(D2);
figure,plot(Dvector1)
figure,plot(Dvector4f)
```
